# Interest in and use of person-centred pharmacy services - a Swiss study of people with diabetes

**DOI:** 10.1186/s12913-021-06217-6

**Published:** 2021-03-10

**Authors:** Noura Bawab, Emilie Zuercher, Tania Carron, Léonie Chinet, Olivier Bugnon, Jérôme Berger, Isabelle Peytremann-Bridevaux

**Affiliations:** 1 Community Pharmacy, Center for Primary Care and Public Health (Unisanté), University of Lausanne, Rue du Bugnon 44, 1011 Lausanne, Switzerland; 2 School of Pharmaceutical Sciences, University of Geneva, Rue Michel-Servet 1, 1211 Geneva 4, Switzerland; 3 Institute of Pharmaceutical Sciences of Western Switzerland (ISPSO), University of Geneva, University of Lausanne, Rue Michel-Servet 1, 1211 Geneva 4, Switzerland; 4 Department of Epidemiology and Health Systems (DESS), Center for Primary Care and Public Health (Unisanté), University of Lausanne, Route de la Corniche 10, 1010 Lausanne, Switzerland; 5 diabètevaud, Avenue de Provence 12, 1007 Lausanne, Switzerland

**Keywords:** Diabetes, Patient support, Pharmacy services, Primary care, Switzerland

## Abstract

**Background:**

Diabetes is one of the most important chronic diseases and affects 9% of the world’s population. To support these people in the day-to-day management of their treatments, pharmacies can offer professional pharmacy services. These are defined as one or more actions organized or provided in a pharmacy to optimize the process of care, with the goal of improving health outcomes and the value of healthcare. Such services have to be tailored to the needs and interests of patients. This study aimed to evaluate interest in and use of pharmacy services among people with diabetes in the canton of Vaud, Switzerland.

**Methods:**

This cross-sectional study analysed self-reported data from 790 people with diabetes included in the CoDiab-VD cohort. Questions focused on sociodemographic and economic characteristics, diabetes and its management, and interest in and use of pharmacy services related to (1) medication intake and adherence and (2) diabetes and general health. Descriptive analyses were first conducted. Logistic regression analyses were then performed for pharmacy services that were of interest to ≥50% of respondents.

**Results:**

The mean age of participants was 66 years, and the sample included more males (59%) than females. The pharmacy services that interested the most respondents were individual interview, pill boxes or weekly pill boxes, treatment plans, checks of all medications, first medical opinions from pharmacists and counselling on devices. Factors significantly associated with interest in pharmacy services were being older, having a lower self-efficacy score, taking more than three medications and having a positive opinion about pharmacists.

**Conclusions:**

This study provides key information on interest in and use of pharmacy services among patients with diabetes in Switzerland; it should help pharmacists individualize their services for patients.

**Supplementary Information:**

The online version contains supplementary material available at 10.1186/s12913-021-06217-6.

## Background

Diabetes is one of the most important chronic diseases and contributes to mortality, morbidity and socio-economic impacts [1]. Worldwide, it affects 9.3% of the population, equal to approximately 463 million people [2]. According to the International Diabetes Federation, the number of people with diabetes will continue to increase over the next decades [3]. To support these people in the daily management of their treatments, pharmacies can offer professional pharmacy services tailored to the needs and interests of patients. The provision of medicines to patients is not sufficient on its own to achieve treatment goals, professional pharmacy services are needed to enable pharmacists to address medication-related needs [4].

Moullin et al. defined a professional pharmacy service as “an action or set of actions undertaken in or organised by a pharmacy, delivered by a pharmacist or other health practitioner, who applies their specialised health knowledge personally or via an intermediary, with a patient or client, population or other health professional, to optimise the process of care, with the aim to improve health outcomes and the value of healthcare” [5].

To our knowledge, little data are available on interest in pharmacy services among people with diabetes. If people with diabetes have no interest in services offered by pharmacies, these will not been used and there would be no benefit to public health. This study aimed to assess interest in and use of pharmacy services among people with diabetes included in the Cohort of Patients with Diabetes in the Canton of Vaud (CoDiab-VD cohort) who responded to the 2017 annual questionnaire, which included a thematic module about pharmacy services [6].

## Methods

### Study design

Data from a cross-sectional survey conducted in the fall of 2017 as part of the CoDiad-VD cohort were used [6]. STROBE (Strengthening the Reporting of Observational Studies in Epidemiology) guidelines were used in the project’s execution and in the manuscript’s preparation [7]. This study is registered with ClinicalTrials.gov, identifier NCT01902043.

### Setting and participants

In Switzerland, community pharmacies can provide pharmacy services, some of which are remunerated and covered for patients by basic health insurance according to the tariff headings [8]. These pharmacy services include pharmacists’ basic cognitive services (e.g., medication delivery, counselling services, prescription/dosage/drug-drug interaction checks, and checks of patient records), medication intake support (directly observed therapy, fractioned delivery or provision of a pill box filled with medication for one or more weeks), or individual interview with the pharmacist [8]. In 2016, the Swiss Federal Council was invited to explore the various possibilities for repositioning pharmacists in primary care. The council stated that community pharmacists have an important role to play and that shifting from traditional medication delivery and counselling towards the provision of patient-centred and interprofessional pharmacy services was essential [9].

In 2011–12 and 2017, people with diabetes were recruited into the CoDiab-VD cohort. Participation in the CoDiab-VD cohort was offered to individuals visiting a participating pharmacy with a diabetes-related prescription. At the time of recruitment, non-institutionalised adults (≥18 years old) who had been diagnosed with diabetes for at least 12 months and were living in the canton of Vaud (French-speaking part of Switzerland) were eligible. Women with gestational diabetes and individuals with cognitive impairment or without sufficient French language skills to complete the questionnaire were excluded [6, 10]. In 2017, the questionnaire was sent by mail to the participants recruited in 2011–12 and were distributed in-person in community pharmacies during the 2017 recruitment period.

Participants who were recruited in 2011–12 and 2017 were very similar at the time of recruitment in terms of their sociodemographic characteristics, health status and behaviours and self-reported diabetes status [11]. In 2017, the total number of respondents who answered to the questionnaire was 790, including 276 individuals included in the CoDiab-VD cohort who were recruited in 2011–12 and 514 newly recruited participants.

### Study questionnaire and data collection

Participants completed a self-administered paper questionnaire that included questions on different aspects of diabetes and diabetes care, questions on their own characteristics, and a thematic module about pharmacy services (see Additional File 1). The participants completed the questionnaire at home and sent it back by mail to the investigators. Participants were free to not answer certain questions.

### Measurements

For the purpose of this study, data collected on the participants’ interest in and use of pharmacy services offered by community pharmacies were used (*very interested, a little interested* or *not interested* and *already used* or *never used*). Two types of pharmacy services were studied: (1) patient support in the management of their medication intake and adherence (individual interview with the pharmacist, SMS or email reminders for medication intake, a smartphone application, (electronic) pill boxes or weekly pill boxes, and treatment plans for all medications); (2) patient support in the management of their diabetes and general health (screening for chronic conditions, monitoring of blood levels or pressure, influenza immunisation, counselling on the use of devices, support to quit smoking or lose weight, first medical opinions from pharmacists about health status, and checks of all medications). Moreover, the participants’ interest in and use of consultation with their reference physicians were investigated.

We also used data collected on participants’ sociodemographic and economic characteristics, including age, sex, education (*primary* - completion of compulsory school or less, *secondary* - vocational training or high school, or *tertiary* - university or technical college); financial hardship affecting participants’ ability to pay household bills during the last 12 months [12]; health status, including perceived health status (first question of the SF-12 questionnaire) [13] and body mass index (kg/m^2^); health behaviours, including physical activity (using questions from the Swiss Health Survey) [14], smoking status and alcohol consumption (using the AUDIT-C questionnaire) [15]; type of diabetes (*type 1, type 2, other* or *unknown*); medication management, including the frequency of pharmacy visits (*≥1 time per week, 2–3 times per month, 1 time per month,* or *< 1 time per month*), the number of medications per day (*1–3, 4–6, 7–9, ≥10 medications*) and the mode of administration of antidiabetic medication (*with* or *without insulin or other injectable drugs*); diabetes self-management, including participation in diabetes education courses; and self-efficacy according to the Stanford Diabetes Self-efficacy scale [16]. Participants’ opinions (*agree*, *disagree*) about their medications and pharmacists were also investigated. Participants’ opinions about their medications were investigated with three questions (derived from the Adherence Estimator, a three-item proximal screener for the likelihood of non-adherence to prescription medications) [17]: 1. I am convinced of the importance of the medications prescribed to me; 2. unreimbursed expenses for medications prescribed to me are a financial burden; 3. I fear that the medications prescribed to me will do me more harm than good. A composite variable for opinion about medications was constructed: respondents who answered respectively (*strongly* or *somewhat*) *agree*, *disagree*, and *disagree* to the three above mentioned questions were considered to have a positive opinion about their medications. Participants’ opinions about pharmacists were also investigated with three questions: 1. pharmacists are health professionals, just like physicians and nurses; 2. pharmacists are experts in medications, side effects and medication interactions; 3. pharmacists are just shopkeepers who sell products in pharmacies. A composite variable for opinion about pharmacists was constructed: respondents who answered respectively (*strongly* or *somewhat*) *agree*, *agree*, and *disagree* to the three above mentioned questions were considered to have a positive opinion about pharmacists. The original questions in French and their English translations are available in the Additional File 1.

### Statistical analyses

First, descriptive analyses were conducted to describe participants’ characteristics, diabetes status (medications and management), opinions about medications and pharmacists, and interest in and use of both types of pharmacy services. Then, multivariate logistic regression analyses were performed to examine which factors were associated with interest in both types of pharmacy services, targeting items that interested at least 50% of the respondents. The following covariates were considered based on their a priori likelihood of influencing interest in pharmacy services: age, sex, education, financial hardship, antidiabetic medication including injections, participation in diabetes education courses, Stanford Diabetes Self-efficacy score, number of medications taken per day (1–3, 4–6, and ≥ 7 medications per day, with the latter category divided between 7 and 9 and ≥ 10), positive opinion about medications, and positive opinion about pharmacists. Odds ratios, predicted probabilities and their 95% confidence intervals were estimated. Moreover, the predicted probabilities of being interested in pharmacy services were plotted according to the number of medications taken per day and the age of the participants, which are patient characteristics known by pharmacists; sex was not present in the graphics because of the absence of a difference in levels of interest between females and males. All other covariates in the logistic regression models were held constant. Logistic regression models were assessed for influential observations and tested their calibration using the Hosmer-Lemeshow goodness-of-fit test. All statistical analyses were performed using Stata 16.0 for Windows (Stata Corporation, College Station, TX, USA, http://www.stata.com). *P*-values < 0.05 were considered statistically significant.

## Results

Table 1 details the sociodemographic characteristics, health status and health behaviours of participants. The mean age of the 790 participants was 66.0 years (range: 18 to 92 years), and the majority of participants were men (59%). Among the participants, 53.2% reported having a secondary education, and 32% reported having difficulty paying bills during the past 12 months. More than the half of the participants (54%) were considered physically active, and 80% were overweight or obese.

**Table 1:** Participants’ characteristics (sociodemographic characteristics, health status and health behaviours)

Variable	N total^e^	% (N) or mean (SD)
**Sociodemographic and economic characteristics**		
Age	790	66.0 (12.5)
Sex	790	
Female		40.9% (323)
Male		59.1% (467)
Education	745	
Primary		15.8% (118)
Secondary		53.2% (396)
Tertiary		31.0% (231)
Financial hardship^a^	768	
Yes		32.4% (249)
No		67.6% (519)
**Health status**		
Perceived health status^b^	779	
Excellent		1.8% (14)
Very good		12.8% (100)
Good		62.8% (489)
Fair		19.9% (155)
Poor		2.7% (21)
BMI (kg/m^2^)	758	
Underweight (< 15.5)		0.7% (5)
Normal (18.5–24.9)		19.7% (149)
Overweight (25–29.9)		38.0% (288)
Obese (≥ 30)		41.7% (316)
**Health behaviours**		
Physical activity^c^	766	
Active		53.7% (411)
Partly active		17.5% (134)
Inactive		28.9% (221)
Smoking status	766	
Non-smoker		39.0% (299)
Former smoker		42.2% (323)
Current smoker		18.8% (144)
Alcohol consumption	752	
Not risky or not excessive		58.0% (462)
Risky or excessive^d^		42.0% (313)

*BMI* Body mass index

^a^ Difficulty paying bills in the last 12 months

^b^ First question of the Short Form Health Survey −12 (SF-12)

^c^ Swiss Health Survey: active: ≥ 150 min of moderate physical activity or ≥ two intense activities per week; partly active: 30 to 149 min of moderate physical activity or one intense activity per week; inactive: < 30 min of moderate physical activity and < one intense activity per week

^d^ Alcohol Use Disorders Identification Test-Consumption (AUDIT-C) score ≥ 4 for men and 3 for women

^e^ The total number of respondents for each item varies since data were collected through a self-administered paper questionnaire and participants were free to not answer certain questions

Most participants (72%) reported having type 2 diabetes, and more than half of participants (57%) received antidiabetic treatment including insulin or another injectable. Most respondents (71%) took more than three medications per day. Details of the frequency of pharmacy visits, diabetes self-management, and participants’ opinions about medications and pharmacists are presented in Table 2.

**Table 2:** Medication management, diabetes self-management, and participants’ opinions about their medications and pharmacists

Variable	Total N^e^	%^a^ (N) or mean (SD; min-max)
**Medication management**		
Type of diabetes	790	
Type 1		11.4% (90)
Type 2		72.0% (569)
Other or unknown		16.6% (131)
Frequency of pharmacy visits	747	
≥ 1 time per week		6.7% (50)
2–3 times per month		30.1% (225)
1 time per month		35.3% (264)
< 1 time per month		27.8% (208)
Number of medications per day	773	
1–3 medications		29.4% (227)
4–6 medications		41.9% (324)
7–9 medications		19.7% (152)
≥ 10 medications		9.1% (70)
Antidiabetic medication	788	
Excluding insulin or other injectables		43.0% (339)
Including insulin or other injectables		57.0% (445)
**Diabetes self-management**		
Participation in one or more diabetes education courses	771	
Yes		35.4% (273)
No		64.6% (498)
Stanford Diabetes Self-efficacy overall score^b^	755	7.5 (1.8; 2.1–10.0)
**Participants’ opinions about their medications**		
“Medications that are prescribed to me are important”	768	
Disagree		4.2% (32)
*Agree*		95.8% (736)
“I fear that prescribed medication are more harmful than beneficial”	757	
*Disagree*		86.4% (654)
Agree		13.6% (103)
“Non-reimbursed medications are burdensome for me”	760	
*Disagree*		27.9% (212)
Agree		72.1% (548)
Positive opinion about medications on all 3 items^c^	770	
*Yes*		23.6% (182)
No		76.4% (588)
**Participants’ opinions about pharmacists**		
“Pharmacists are experts in medications, side effects and medication interactions”	741	
Disagree		6.3% (47)
*Agree*		93.7% (694)
“Pharmacists are health professionals, just like physicians or nurses”	734	
Disagree		15.7% (115)
*Agree*		84.3% (619)
“Pharmacists are just shopkeepers who sell products in pharmacy”	722	
*Disagree*		85.9% (620)
Agree		14.1% (102)
Positive opinion about pharmacists on all 3 items^d^	764	
*Yes*		65.5% (500)
No		34.6% (264)

^a^ Due to rounding, the sum of the percentages is not always equal to 100%

^b^ The Stanford Diabetes Self-efficacy overall score ranges from 0 to 10, with a higher score indicating a higher level of self-efficacy

^c^ Composite variable for opinion about medication: respondents answering *agree*, *disagree*, and *disagree* to the three items, in that order, were considered to have a positive opinion

^d^ Composite variable for opinion about pharmacists: respondents answering *agree*, *agree*, and *disagree* to the three items, in that order, were considered to have a positive opinion

^e^ The total number of respondents for each item varies since data were collected through a self-administered paper questionnaire and participants were free to not answer certain questions

**Fig. 1:**
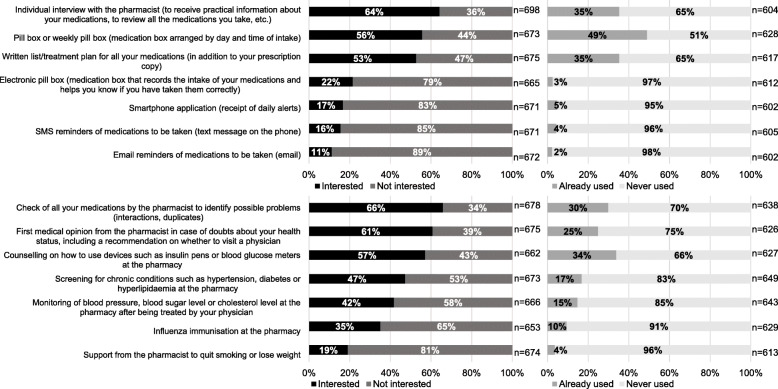
Pharmacy services interest and utilisation: medication intake and adherence (top), diabetes and general health (bottom)

The proportions of participants who were interested in different pharmacy services and who declared having previously used them are presented in Fig. 1. Pharmacy services that generated the greatest interest were also those that were the most used: individual interview, pill boxes, treatment plans, checks of all medications, first medical opinions and counselling on devices. In addition, 85% of the respondents were interested in receiving practical information about their medications during a medical consultation with their physician, and 59% declared that they already benefited from this service (data not illustrated).

The results of logistic regression analyses of the pharmacy services that interested at least 50% of the participants are presented in Table 3; the predicted probabilities are available in the Additional File 2.

**Table 3:** Logistic regression analyses of the pharmacy services that interested ≥50% of the participants

		Medication intake and adherence						Diabetes and general health					
		Interview with pharmacist (***n*** = 625)		Pill box or weekly pill box (***n*** = 608)		List of all medications/treatment plan (***n*** = 606)		Check of all medications (***n*** = 610)		First medical opinion (n = 608)		Counselling on how to use devices (***n*** = 596)	
		OR (95% CI)	p-value	OR (95% CI)	p-value	OR (95% CI)	p-value	OR (95% CI)	p-value	OR (95% CI)	p-value	OR (95% CI)	p-value
**Age**	< 65 years	1.00		1.00		1.00		1.00		1.00		1.00	
	65–74 years	2.03 (1.35 to 3.04)	**< 0.01**	1.26 (0.84 to 1.88)	0.26	1.26 (0.84 to 1.89)	0.26	0.93 (0.62 to 1.42)	0.75	0.82 (0.55 to 1.22)	0.33	1.36 (0.91 to 2.03)	0.14
	≥75 years	3.28 (1.95 to 5.52)	**< 0.01**	2.05 (1.24 to 3.37)	**0.01**	1.41 (0.87 to 2.30)	0.16	0.69 (0.42 to 1.12)	0.13	0.51 (0.32 to 0.82)	**0.01**	1.01 (0.63 to 1.61)	0.97
**Sex**	Female	1.00		1.00		1.00		1.00		1.00		1.00	
	Male	1.10 (0.76 to 1.59)	0.60	0.90 (0.63 to 1.30)	0.58	1.22 (0.85 to 1.75)	0.29	0.84 (0.58 to 1.22)	0.35	0.69 (0.48 to 1.00)	0.05	0.69 (0.48 to 1.00)	0.05
**Education**	Primary	1.00		1.00		1.00		1.00		1.00		1.00	
	Secondary	0.93 (0.54 to 1.59)	0.79	0.79 (0.46 to 1.34)	0.38	1.16 (0.69 to 1.96)	0.58	1.65 (0.98 to 2.78)	0.06	1.08 (0.64 to 1.82)	0.77	1.22 (0.72 to 2.08)	0.45
	Tertiary	0.76 (0.43 to 1.34)	0.34	0.91 (0.51 to 1.62)	0.75	1.14 (0.65 to 2.02)	0.65	1.95 (1.10 to 3.46)	**0.02**	1.33 (0.76 to 2.35)	0.32	0.99 (0.56 to 1.75)	0.98
**Financial hardship**	No	1.00		1.00		1.00		1.00		1.00		1.00	
	Yes	1.38 (0.93 to 2.06)	0.11	1.21 (0.82 to 1.79)	0.34	1.21 (0.82 to 1.79)	0.33	1.11 (0.74 to 1.66)	0.61	1.37 (0.92 to 2.03)	0.12	1.13 (0.77 to 1.67)	0.53
**Treatment including injections**	No	1.00		1.00		1.00		1.00		1.00		1.00	
	Yes	1.02 (0.71 to 1.48)	0.91	1.12 (0.78 to 1.61)	0.53	1.09 (0.76 to 1.57)	0.63	0.79 (0.55 to 1.15)	0.22	1.05 (0.74 to 1.51)	0.77	1.25 (0.88 to 1.78)	0.22
**Participation in diabetes education course**	No	1.00		1.00		1.00		1.00		1.00		1.00	
	Yes	1.03 (0.71 to 1.51)	0.87	0.85 (0.59 to 1.23)	0.38	0.74 (0.51 to 1.08)	0.12	1.24 (0.85 to 1.83)	0.27	1.04 (0.72 to 1.51)	0.82	0.80 (0.55 to 1.15)	0.23
**Stanford Diabetes Self-efficacy**	Overall score	0.94 (0.85 to 1.04)	0.23	0.88 (0.79 to 0.97)	**0.01**	0.88 (0.79 to 0.97)	**0.01**	0.90 (0.81 to 1.00)	0.06	0.87 (0.79 to 0.97)	**0.01**	0.90 (0.81 to 0.99)	**0.03**
**Number of medications taken**	1 to 3	1.00		1.00		1.00		1.00		1.00		1.00	
	4 to 6	1.51 (1.00 to 2.26)	**0.05**	2.10 (1.40 to 3.15)	**< 0.01**	1.84 (1.23 to 2.77)	**< 0.01**	1.29 (0.85 to 1.94)	0.23	0.94 (0.62 to 1.41)	0.75	1.14 (0.76 to 1.71)	0.52
	≥7	2.06 (1.27 to 3.35)	**< 0.01**	3.47 (2.16 to 5.57)	**< 0.01**	3.84 (2.39 to 6.18)	**< 0.01**	2.11 (1.28 to 3.48)	**< 0.01**	1.20 (0.74 to 1.93)	0.46	1.28 (0.80 to 2.05)	0.30
**Positive opinion about medication**	Less positive	1.00		1.00		1.00		1.00		1.00		1.00	
	Positive +++	0.95 (0.62 to 1.46)	0.83	0.92 (0.61 to 1.40)	0.71	0.74 (0.48 to 1.12)	0.16	0.90 (0.59 to 1.39)	0.64	1.23 (0.81 to 1.88)	0.33	0.84 (0.55 to 1.27)	0.41
**Positive opinion about pharmacists**	Less positive	1.00		1.00		1.00		1.00		1.00		1.00	
	Positive +++	1.69 (1.17 to 2.45)	**0.01**	1.32 (0.91 to 1.91)	0.15	1.21 (0.84 to 1.76)	0.30	1.58 (1.09 to 2.28)	**0.02**	1.51 (1.05 to 2.17)	**0.03**	1.46 (1.02 to 2.10)	**0.04**

Significant p-values (< 0.05) in bold

Higher age, tertiary education, lower self-efficacy score, taking more than three medications, and a positive opinion about pharmacists were all significantly associated with interest in certain pharmacy services. A lower self-efficacy score was significantly associated with greater interest in pill boxes, treatment plans, first medical opinions, and counselling on device use. Taking more than three medications was associated with greater interest in individual interview with the pharmacist, pill boxes, and treatment plans, while taking ≥7 medications was associated with interest in checks of all medications. Participants who reported a positive opinion about pharmacists were more interested in individual interview with the pharmacist, checks of all medications, first medical opinions, and counselling on device use. Sex, financial hardship, diabetes treatment including injections, participation in diabetes education courses, and a positive opinion about medication were not significantly associated with interest in any of the pharmacy services investigated.

Based on the logistic regression models, predicted probabilities of being interested in pharmacy services were computed according to number of medications taken and age, with all other covariates held constant; the results are presented in the Additional File 3. The older participants were and the more medications they took, the more they were interested in services related to medication intake and adherence. For services related to diabetes and general health, the trends were less clear; a higher number of medications taken was associated with higher probabilities of interest, while older age was associated with lower probabilities of interest in those services.

## Discussion

This study describes interest in and use of pharmacy services among Swiss patients with diabetes in the CoDiab-VD cohort. The pharmacy services that interested the most respondents were individual interview, pill boxes, treatment plans, checks of all medications, first medical opinions from pharmacists and counselling on devices. According to the participants, the most valuable pharmacy services related to medication intake and adherence as well as diabetes and general health were mainly personal and patient-specific, which highlights the need to individualise and target specific services based on patients’ personal needs. Furthermore, first medical opinions and checks of all medications were the two services with the greatest differences between interest and use levels, indicating an opportunity to develop these services to meet patients’ needs.

Factors positively associated with interest in pharmacy services were higher age, higher education level, taking four or more medications and a positive opinion about pharmacists, while the self-efficacy score was negatively associated with interest in pharmacy services. Participants’ opinions about their medications mainly showed that their prescribed medications are important to them and that they do not fear of harm from the medications for 96 and 86% of respondents, respectively. Nearly three quarters of respondents were rather concerned about the non-reimbursement of medications. This finding probably reflects a general concern about medications, their prices and their reimbursement rather than a specific concern related to antidiabetic medications, as these medications are all reimbursed in the Swiss health system; the finding also shows the need to propose pharmacy services that are reimbursed. In addition, medications can be dispensed to patients by pharmacies for up to three months to be reimbursed by health insurances, requiring frequent visits to the pharmacy. This gives pharmacists the opportunity to play a key role in providing pharmaceutical support to the patient at each pharmacy visit.

Pharmacists are very accessible health care professionals, as most of the respondents visited a pharmacy at least once a month. This accessibility combined with the positive opinion about pharmacists suggests that pharmacists could actively participate in quality improvement initiatives targeting the care of patients with chronic conditions.

Multivariate analyses showed that the most notable factors related to interest in pharmacy services were being older, having a lower self-efficacy score, taking more than three medications and having a positive opinion about pharmacists. Lower Stanford self-efficacy scores mean that participants are less confident about being able to overcome barriers and accomplish tasks. Lower self-efficacy scores in this study were related to higher interest in pharmacy services, which may have been related to patients’ beliefs about the need for pharmacy services. When patients perceive the need for and benefits of pharmacy services, they are more interested in them [18]. In contrast, patients who believe they do not need pharmacy services [19–21] and who are satisfied with their current medication [19] logically have a lower interest in, or use of, pharmacy services. Taking more than three medications per day was associated with greater interest in certain pharmacy services, confirming that taking more medications is associated with a greater number of drug-related problems, which is a measure of the potential value of (interest in and use of) pharmacy services from patients’ points of view [22, 23]. Moreover, having a positive opinion about pharmacists can indicate an appreciated personal relation with the pharmacist based on good communication [21]. Seeing the pharmacist as a trusted and accessible expert in his or her area of expertise is also associated with increased interest in the use of pharmacy services [20]. In Switzerland, this association has been identified by the national government, which financially supports the scientific evaluation of the implementation of an interprofessional and tailored support programme (safety and medication adherence) for people with type 2 diabetes [9, 24].

The main strength of this study was that the survey included people with diabetes spread throughout a Swiss region who were recruited from community pharmacies. This approach should have allowed the inclusion of participants who were more representative of the population of patients with diabetes than if the recruitment had been carried out in a specialised medical or hospital setting.

In the interpretation of the results, the following limitations need to be considered. Data were based exclusively on self-reports, which involves the probable over- or under-representation of certain phenomena. Without access to other data, however, the use of this type of data is considered appropriate [25]. The recruitment method allowed us to limit selection bias; the limited selection bias was also supported by the fact that the characteristics of the participants in this cohort were comparable to those of people with diabetes in other Swiss studies in terms of age, sex, smoking status, body mass index, and total number of medications taken [26–29].

## Conclusion

The results of this study provide a better understanding of the people who are most interested in pharmacy services to support the assessment of their needs and the development of tailored, appropriate solutions. These results should also motivate pharmacists to explain the importance of pharmacy services so that people can perceive their benefits. Since pharmacies are often visited by patients with chronic conditions, more effort should be made to involve pharmacists in health promotion or prevention initiatives such as flu vaccination and weight loss or smoking cessation programmes.

## Supplementary Information

**Additional file 1.** Participant questionnaire items. Original items of the participant questionnaire in French and their translations into English.

**Additional file 2.** Predicted probabilities obtained from logistic regression analyses of the pharmacy services that interested ≥50% of the participants. Predicted probabilities obtained from logistic regression analyses of the pharmacy services that interested ≥50% of the participants and their 95% confidence intervals.

**Additional file 3.** Predicted probabilities of interest in pharmacy services according to number of medications and age with all other covariates held constant in the logistic regression models. Predicted probabilities of interest in pharmacy services according to number of medications (1–3, 4–6 or ≥ 7 medications) and age (< 65, 65–74, ≥75 years) and with all other covariates held constant in the logistic regression models.

## Data Availability

The metadata from the CoDiab-VD datasets supporting the conclusions of this article are available in a public repository (CoDiab-VD: Cohort of Patients with Diabetes in the Canton of Vaud (Switzerland)), 10.16909/dataset/18. Data are available upon request to be made via the repository.

## Abbreviations

CoDiab-VD: Cohort of Patients with Diabetes in the Canton of Vaud
STROBE: Strengthening the Reporting of Observational Studies in Epidemiology
