# Supplementary material for: Interest in and use of person-centred pharmacy services - a Swiss study of people with diabetes
Source: BMC Health Serv Res. 2021 Mar 10;21:216. doi: 10.1186/s12913-021-06217-6 (PMC7945663; doi:10.1186/s12913-021-06217-6)
Supplement: Supplementary file 1 — Additional file 1. Participant questionnaire items. Original items of the participant questionnaire in French and their translations into English. [file 12913_2021_6217_MOESM1_ESM.docx]

# Additional File 1. Participant questionnaire items

| French (original) | English (translation) |
| --- | --- |
| Sociodemographic and economic characteristics | |
| Laquelle des propositions suivantes décrit le mieux la formation la plus élevée que vous avez terminée ?  - Aucune scolarité achevée  - Scolarité obligatoire  - Apprentissage (CFC), formation/école professionnelle achevée  - Maturité (baccalauréat), maturité professionnelle, école normale, école de commerce, école de culture générale  - Ecole technique et/ou professionnelle supérieure, maîtrise fédérale ou professionnelle  - Niveau universitaire (y compris Hautes Ecoles Spécialisées (HES), Ecole polytechnique, Beaux Arts, etc.)  - Autre | Which of the following best describes the highest training you have completed?  - No schooling completed  - Compulsory education  - Initial vocational training (Federal Certificate of Capacity) or professional training or school completed  - General or professional baccalaureate, teacher training school, or upper secondary specialised school  - Technical and/or higher vocational school or Federal Diploma of Higher Education and Advanced Federal Diploma of Higher Education  - University level (including specialised high schools, polytechnic schools, fine arts schools, etc.)  - Other |
| Durant les 12 derniers mois, avez-vous eu de la peine à payer les factures de votre ménage (impôts, assurances, téléphone, électricité, carte de crédit, etc.) ?  Oui ou non | During the last 12 months, have you had trouble paying your household bills (taxes, insurance, telephone, electricity, credit cards, etc.)?  Yes or No |
| Health status | |
| Quel est votre poids actuel ? (en kg) | What is your current weight? (in kg) |
| Quelle est votre taille actuelle (hauteur) ? (en cm) | What is your current height? (in cm) |
| Health behaviours | |
| Mettez une croix dans la case qui vous décrit le mieux  - Je fume actuellement  - J'ai fumé mais je ne fume plus actuellement  - Je n'ai jamais fumé | Check the box that best describes you:  - I am a smoker  - I used to smoked but I no longer smoke  - I have never smoked |
| Pendant vos loisirs, avez-vous au moins une fois par semaine une activité physique qui vous fasse transpirer ? (par exemple : course à pied, vélo, marche rapide…)  Oui ou non  Si oui, en moyenne, combien de jours par semaine ? | In your spare time, do you engage at least once a week in a physical activity that makes you sweat? (e.g., running, cycling, brisk walking...)  Yes or No  If yes, how many days per week on average? |
| Les questions suivantes ne concernent plus seulement les activités physiques qui vous font transpirer, mais également  d'autres formes de mouvements moins intensifs. | The following questions no longer only concern physical activities that make you sweat but also other forms of less intensive activity. |
| Si vous pensez à des activités physiques au cours desquelles vous êtes au moins un peu essoufflé(e), comme la marche rapide, les excursions à pied, la danse, le jardinage ou différents sports: combien de jours par semaine pratiquez-vous de telles activités physiques ?  - Jours par semaine *(de 1 à 7 jours)*  - Jamais  - Je ne sais pas | Thinking about physical activities in which you are at least a little out of breath, such as brisk walking, hiking, dancing, gardening or various sports, how many days per week do you do such physical activities?  - Days per week (1 to 7 days)  - Never  - I don't know |
| Quelle est en moyenne la durée de ces activités physiques pendant ces jours ?  - Heures et minutes par jour  - Je ne sais pas | What is the average duration of such physical activity on these days?  - Hours and minutes per day  - I don't know |
| Medication management | |
| Savez-vous de quel type de diabète vous êtes atteint(e) ?  - Type 1  - Type 2  - Autre  - Je ne sais pas | Do you know what type of diabetes you have?  - Type 1  - Type 2  - Other  - I don’t know |
| A quelle fréquence vous rendez-vous en pharmacie pour aller chercher vos médicaments ?  - Une fois par semaine ou plus souvent  - 2-3 fois par mois  - Une fois par mois  - Moins d’une fois par mois | How often do you go to the pharmacy to get your medication?  - Once a week or more often  - 2-3 times a month  - Once a month  - Less than once a month |
| Combien de médicaments différents prenez vous par jour ? *(si vous prenez plusieurs fois le même médicament, comptez-le une seule fois)*  - 1 à 3 médicaments par jour  - 4 à 6 médicaments par jour  - 7 à 9 médicaments par jour  - 10 médicaments par jour ou plus  - Je ne prends aucun médicament  - Je ne sais pas | How many different medications do you take per day? *(if you are taking the same medication several times per day, count it only once)*  - 1 to 3 medications per day  - 4 to 6 medications per day  - 7 to 9 medications per day  - 10 or more medications per day  - I don't take any medication  - I don't know |
| Actuellement, vous êtes traité(e) pour le diabète par… ? *(plusieurs réponses possibles)*  - Des comprimés (antidiabétiques oraux)  - De l’insuline  - Une injection autre que de l'insuline (par exemple : Victoza, Byetta, Bydureon)  - Aucun des traitements mentionnés  - Je ne sais pas | Currently, you are being treated for diabetes with...? (*multiple answers allowed*)  - Tablets (oral antidiabetic medication)  - Insulin  - An injection other than insulin (e.g., Victoza, Byetta, Bydureon)  - None of the treatments mentioned  - I don't know |
| Diabetes self-management | |
| Depuis le diagnostic de votre diabète, avez-vous participé à un ou des cours pour patients sur la gestion de votre diabète (séances individuelles ou en groupe) ? *(plusieurs réponses possibles)*  - Oui, j’ai participé dans l’année qui a suivi mon diagnostic  - Oui, j’ai participé plus d’une année après mon diagnostic  - Oui, j’ai participé à la suite d’une hospitalisation  - Oui, j’ai participé à la suite d’une complication de mon diabète (sans hospitalisation)  - Oui, j’ai participé à un cours spécifique sur la gestion de l’insuline lors de l’introduction du traitement par insuline  - Non, je n'ai jamais participé à un cours sur la gestion de mon diabète  - Je ne sais pas | Since you were diagnosed with diabetes, have you attended any diabetes education courses (either one-on-one or group sessions)? (*multiple answers allowed*)  - Yes, I attended in the year following my diagnosis  - Yes, I attended more than a year after my diagnosis  - Yes, I attended following a hospitalisation  - Yes, I attended following a diabetes-related complication (without hospitalisation)  - Yes, I attended a specific course on the management of insulin use when an insulin therapy was introduced  - No, I have never attended any diabetes education course  - I don’t know |
| People’s opinions about their medications and pharmacists | |
| Option de réponses  - Pas du tout d’accord  - Plutôt pas d’accord  - Plutôt d’accord  - Tout à fait d’accord  - Sans avis *(première question uniquement)* | Response options  - Strongly disagree  - Somewhat disagree  - Somewhat agree  - Strongly agree  - No opinion (*first question only)* |
| Veuillez indiquer votre degré d'accord ou de désaccord avec les affirmations suivantes à propos de votre traitement médicamenteux :  - Je suis convaincu(e) de l'importance des médicaments qui me sont prescrits  - Les frais non remboursés pour les médicaments qui me sont prescrits sont un poids financier pour moi  - Je crains que les médicaments qui me sont prescrits me fassent plus de mal que de bien | Please indicate your level of agreement or disagreement with the following statements about your mediations:  -I am convinced of the importance of the medications prescribed to me  -Unreimbursed expenses for medications prescribed to me are a financial burden  -I fear that the medications prescribed to me will do me more harm than good |
| Veuillez indiquer votre degré d'accord ou de désaccord avec les affirmations ci-dessous à propos du rôle des pharmaciens :  - Les pharmaciens sont des professionnels de santé, au même titre que les médecins et les infirmiers(ères)  - Les pharmaciens sont des experts en médicaments, effets secondaires et interactions médicamenteuses  - Les pharmaciens sont juste des commerçants qui vendent des produits en pharmacie | Please indicate your level of agreement or disagreement with the following statements about the role of pharmacists:  - Pharmacists are health professionals, just like physicians and nurses  - Pharmacists are experts in medications, side effects and medication interactions  -Pharmacists are just shopkeepers who sell products in pharmacies |
| Pharmacy services | |
| Option de réponses  Intérêt personnel :  Non, ça ne m'intéresse pas  Oui, ça m'intéresse un peu  Oui, ça m'intéresse beaucoup  Déjà utilisé ?  Oui, déjà utilisé  Non, jamais utilisé | Response options  Personal interest:  No, I'm not interested  Yes, I'm a little interested  Yes, I'm very interested  Already used?  Yes, already used  No, never used |
| Etes-vous intéressé(e) par les aides suivantes pour vous soutenir dans la prise de vos médicaments, et les avez-vous déjà utilisées ?  - Entretien individuel avec le pharmacien (pour recevoir des informations pratiques sur vos médicaments, faire un bilan de tous les médicaments que vous prenez, etc.)  - Consultation spécifique chez votre médecin (pour recevoir des informations pratiques sur vos médicaments, faire un bilan de tous les médicaments que vous prenez, etc.)  - Rappels des médicaments à prendre par SMS (message texte sur le téléphone)  - Rappels des médicaments à prendre par email (message électronique, courriel)  - Application sur smartphone (réception d'alertes journalières)  - Pilulier ou semainier (boîte à médicaments, rangés par jours et heures de prise)  - Pilulier électronique (boîte à médicaments qui enregistre la prise de vos médicaments et vous aide à savoir si vous les avez bien pris)  - Liste écrite / plan de traitement de tous vos médicaments (en dehors de l'ordonnance) | Are you interested in the following aids to support you in taking your medication, and have you ever used them?  - Individual consultation with the pharmacist (to receive practical information about your medications, to review all the medications you take, etc.)  - Specific consultation with your physician (to receive practical information about your medications, to review all the medications you take, etc.)  - SMS reminders of medications to be taken (text message on the phone)  - Email reminders of medications to be taken (email)  - Smartphone application (receipt of daily alerts)  - Pill box or weekly pill box (medication box arranged by day and time of intake)  - Electronic pill box (medication box that records the intake of your medications and helps you know if you have taken them correctly)  - Written list or treatment plan for all your medications (in addition to your prescription copy) |
| Etes-vous intéressé(e) par les services suivants proposés en pharmacie, et les avez-vous déjà utilisés ?  - Tests en pharmacie pour détecter les maladies chroniques telles que l'hypertension, le diabète ou un niveau de cholestérol trop élevé (dépistage)  - Surveillance en pharmacie de votre pression/tension artérielle, taux de sucre dans le sang, ou taux de cholestérol, après avoir reçu un traitement de votre médecin  - Vaccination contre la grippe en pharmacie  - Conseils en pharmacie sur la manière d'utiliser des appareils, tels que les stylos injecteurs d'insuline ou les lecteurs de glycémie  - Aide par le pharmacien pour arrêter de fumer ou contrôler votre poids  - Premier avis de la part du pharmacien en cas de doute sur votre état de santé, incluant la recommandation de consulter ou non un médecin  - Contrôle de tous vos médicaments par le pharmacien afin de détecter d'éventuels problèmes (interactions, doublons) | Are you interested in the following pharmacy services and have you ever used them?  - Screening for chronic conditions such as hypertension, diabetes or hyperlipidaemia at the pharmacy  - Monitoring of blood pressure, blood sugar level or cholesterol level in pharmacy after being treated by your physician  - Influenza immunisation at the pharmacy  - Counselling on how to use devices such as insulin pens or blood glucose meters at the pharmacy  - Support from the pharmacist to quit smoking or lose weight  - First medical opinion from the pharmacist in case of doubts about your health status, including a recommendation on whether to visit a physician  - Check of all your medications by the pharmacist to identify possible problems (interactions, duplicates) |
