# Supplementary material for: Interest in and use of person-centred pharmacy services - a Swiss study of people with diabetes
Source: BMC Health Serv Res. 2021 Mar 10;21:216. doi: 10.1186/s12913-021-06217-6 (PMC7945663; doi:10.1186/s12913-021-06217-6)
Supplement: Supplementary file 2 — Additional file 2. Predicted probabilities obtained from logistic regression analyses of the pharmacy services that interested ≥50% of the participants. Predicted probabilities obtained from logistic regression analyses of the pharmacy services that interested ≥50% of the participants and their 95% confidence intervals. [file 12913_2021_6217_MOESM2_ESM.docx]

# Additional File 2. Predicted probabilities obtained from logistic regression analyses of the pharmacy services that interested ≥50% of the participants

|  |  | **Medication intake and adherence** | | | | | | **Diabetes and general health** | | | | | |
| --- | --- | --- | --- | --- | --- | --- | --- | --- | --- | --- | --- | --- | --- |
|  |  | **Consultation with pharmacist (n=625)** | | **Pill box or weekly pill box (n=608)** | | **List of all medications/ treatment plan (n=606)** | | **Check of all medications (n=610)** | | **First medical opinion (n=608)** | | **Counselling on how to use devices (n=596)** | |
|  |  | Predicted probability | (95% CI) | Predicted probability | (95% CI) | Predicted probability | (95% CI) | Predicted probability | (95% CI) | Predicted probability | (95% CI) | Predicted probability | (95% CI) |
| **Age** | <65 years | 0.54 | [0.47 to 0.60] | 0.50 | [0.44 to 0.57] | 0.49 | [0.43 to 0.56] | 0.69 | [0.63 to 0.75] | 0.66 | [0.60 to 0.72] | 0.55 | [0.49 to 0.61] |
|  | 65-74 years | 0.69 | [0.63 to 0.75] | 0.56 | [0.49 to 0.62] | 0.55 | [0.48 to 0.61] | 0.68 | [0.61 to 0.74] | 0.61 | [0.55 to 0.68] | 0.62 | [0.56 to 0.69] |
|  | ≥75 years | 0.78 | [0.71 to 0.85] | 0.66 | [0.58 to 0.74] | 0.57 | [0.49 to 0.66] | 0.61 | [0.52 to 0.69] | 0.50 | [0.42 to 0.59] | 0.55 | [0.47 to 0.64] |
| **Sex** | Female | 0.63 | [0.57 to 0.69] | 0.57 | [0.51 to 0.63] | 0.50 | [0.44 to 0.57] | 0.69 | [0.63 to 0.75] | 0.66 | [0.60 to 0.72] | 0.63 | [0.56 to 0.69] |
|  | Male | 0.65 | [0.61 to 0.70] | 0.55 | [0.50 to 0.60] | 0.55 | [0.50 to 0.59] | 0.65 | [0.61 to 0.70] | 0.58 | [0.53 to 0.63] | 0.54 | [0.49 to 0.59] |
| **Education** | Primary | 0.67 | [0.58 to 0.77] | 0.59 | [0.49 to 0.69] | 0.50 | [0.40 to 0.61] | 0.56 | [0.45 to 0.67] | 0.58 | [0.47 to 0.69] | 0.55 | [0.44 to 0.67] |
|  | Secondary | 0.66 | [0.61 to 0.71] | 0.54 | [0.49 to 0.59] | 0.54 | [0.48 to 0.59] | 0.67 | [0.62 to 0.72] | 0.60 | [0.54 to 0.65] | 0.60 | [0.54 to 0.65] |
|  | Tertiary | 0.61 | [0.55 to 0.68] | 0.57 | [0.50 to 0.64] | 0.53 | [0.47 to 0.60] | 0.70 | [0.64 to 0.77] | 0.64 | [0.58 to 0.71] | 0.55 | [0.48 to 0.62] |
| **Financial hardship** | No | 0.62 | [0.58 to 0.67] | 0.54 | [0.49 to 0.59] | 0.52 | [0.47 to 0.56] | 0.66 | [0.61 to 0.71] | 0.59 | [0.54 to 0.64] | 0.57 | [0.52 to 0.61] |
|  | Yes | 0.69 | [0.63 to 0.75] | 0.58 | [0.52 to 0.65] | 0.56 | [0.49 to 0.63] | 0.68 | [0.62 to 0.75] | 0.66 | [0.59 to 0.73] | 0.59 | [0.52 to 0.67] |
| **Treatment including injections** | No | 0.64 | [0.58 to 0.70] | 0.54 | [0.48 to 0.60] | 0.52 | [0.46 to 0.58] | 0.70 | [0.64 to 0.75] | 0.60 | [0.54 to 0.66] | 0.55 | [0.48 to 0.61] |
|  | Yes | 0.65 | [0.60 to 0.69] | 0.57 | [0.52 to 0.62] | 0.54 | [0.49 to 0.59] | 0.65 | [0.60 to 0.70] | 0.62 | [0.56 to 0.67] | 0.60 | [0.55 to 0.65] |
| **Participation in diabetes education course** | No | 0.64 | [0.60 to 0.69] | 0.57 | [0.52 to 0.62] | 0.55 | [0.51 to 0.60] | 0.65 | [0.60 to 0.70] | 0.61 | [0.56 to 0.65] | 0.59 | [0.55 to 0.64] |
|  | Yes | 0.65 | [0.59 to 0.71] | 0.53 | [0.47 to 0.60] | 0.49 | [0.42 to 0.55] | 0.70 | [0.64 to 0.76] | 0.62 | [0.55 to 0.68] | 0.54 | [0.47 to 0.61] |
| **Number of medications taken** | 1 to 3 | 0.56 | [0.49 to 0.64] | 0.40 | [0.33 to 0.47] | 0.38 | [0.31 to 0.45] | 0.60 | [0.53 to 0.67] | 0.61 | [0.53 to 0.68] | 0.55 | [0.47 to 0.62] |
|  | 4 to 6 | 0.65 | [0.60 to 0.71] | 0.58 | [0.52 to 0.64] | 0.53 | [0.46 to 0.59] | 0.66 | [0.60 to 0.71] | 0.59 | [0.53 to 0.65] | 0.58 | [0.52 to 0.64] |
|  | ≥7 | 0.72 | [0.65 to 0.79] | 0.69 | [0.62 to 0.76] | 0.69 | [0.62 to 0.76] | 0.75 | [0.69 to 0.82] | 0.65 | [0.57 to 0.72] | 0.60 | [0.53 to 0.68] |
| **Positive opinion about medication** | Less positive | 0.65 | [0.61 to 0.69] | 0.56 | [0.52 to 0.60] | 0.55 | [0.50 to 0.59] | 0.67 | [0.63 to 0.71] | 0.60 | [0.55 to 0.64] | 0.59 | [0.54 to 0.63] |
|  | Positive +++ | 0.64 | [0.56 to 0.71] | 0.54 | [0.46 to 0.62] | 0.48 | [0.40 to 0.56] | 0.65 | [0.57 to 0.73] | 0.64 | [0.57 to 0.72] | 0.54 | [0.46 to 0.63] |
| **Positive opinion about pharmacists** | Less positive | 0.57 | [0.50 to 0.64] | 0.51 | [0.45 to 0.58] | 0.50 | [0.43 to 0.57] | 0.60 | [0.53 to 0.67] | 0.55 | [0.48 to 0.62] | 0.52 | [0.45 to 0.59] |
|  | Positive +++ | 0.68 | [0.64 to 0.72] | 0.58 | [0.53 to 0.62] | 0.54 | [0.50 to 0.59] | 0.70 | [0.66 to 0.74] | 0.64 | [0.59 to 0.69] | 0.60 | [0.56 to 0.65] |
