# Supplementary material for: Interest in and use of person-centred pharmacy services - a Swiss study of people with diabetes
Source: BMC Health Serv Res. 2021 Mar 10;21:216. doi: 10.1186/s12913-021-06217-6 (PMC7945663; doi:10.1186/s12913-021-06217-6)
Supplement: Supplementary file 3 — Additional file 3. Predicted probabilities of interest in pharmacy services according to number of medications and age with all other covariates held constant in the logistic regression models. Predicted probabilities of interest in pharmacy services according to number of medications (1–3, 4–6 or ≥ 7 medications) and age (< 65, 65–74, ≥75 years) and with all other covariates held constant in the logistic regression models. [file 12913_2021_6217_MOESM3_ESM.docx]

# Additional File 3. Predicted probabilities of interest in pharmacy services according to number of medications and age with all other covariates held constant in the logistic regression models

| **Medication intake and adherence** | **Diabetes and general health** |
| --- | --- |
|  |  |
|  |  |
|  |  |
|  | |
